# A Case of Heteroplastic Ovarian Grafting, Followed by Pregnancy, and the Delivery of a Living Child, with Discussion1Read at a meeting of the Surgical Section of the New York Academy of Medicine, April 6, 1906.

**Published:** 1907-02

**Authors:** Robert T. Morris

**Affiliations:** New York. Professor of Surgery in the New York Post-Graduate Medical School and Hospital; Fellow of the New York Academy of Medicine; Member of American Medical Association, Etc.


### SPECIAL ARTICLE—SELECTED.

### A Case of Heteroplastic Ovarian Grafting, Followed by Pregnancy, and the Delivery of a Living Child, with Discussion.[1]

By ROBERT T. MORRIS, M. D., New York.
Professor of Surgery in the New York Post-Graduate Medical School and Hospital; Fellow of the New York Academy of Medicine; Member of American Medical Association, Etc.

*(Medical Record, May 5, 1906.)*

MRS. H. W. was sent to me at the Post-Graduate Hospital by her physician, Dr. H. L. Barker of Woodside, New York, on February 1, 1902. She was 21 years of age, began menstruating at 15, and stopped menstruating at 19. Previous to the cessation of menstruation the function had been of average character. She was married at the age of 18, became pregnant soon after, and had a miscarriage at the third month. During the two years previous to her entrance into the Post-Graduate Hospital she had suffered from the common menopause symptoms of pelvic pain, headache, and hot and cold flashes. These symptoms had steadily increased in intensity, and were most marked at the times when the monthly flow should have appeared.

I made a diagnosis of cirrhotic ovaritis and asked the patient to remain in the hospital until an opportunity presented for obtaining ovarian grafts from some other patient, to replace her own ovaries, which were to be removed. A week later Mrs. W. was prepared for operation, but as I did not quite like the character of the ovaries from which grafts were to be taken, the patient was asked to wait for a better choice. On February 11, 1902, Dr. H. J. Boldt had occasion to operate upon Mrs. G. P. for uterine prolapse. Mrs. P. was 33 years of age, and the mother of three children. Her ovaries were normal excepting for the congestion common to prolapse cases. While Dr.

1. Read at a meeting of the Surgical Section of the New York Academy of Medicine, April 6, 1906.

Boldt was operating I removed from Mrs. P. ovarian tissue in the form of a wedge-shaped ribbon, not quite as much as usual in my experimental work, but enough to be incidentally beneficial in relieving the congestion for Mrs. P., at the same time that grafts were obtained for Mrs. W. The grafts were placed in physiological saline solution at a temperature of 100° Fahrenheit, and maintained at the temperature by a nurse until the abdomen of Mrs. W. had been opened through a short median incision. (It is important in dealing with delicate structures to choose the saline solution, which is isotonic with human blood).

The ovaries of Mrs. W. were removed with Tuffier's angiotribe. The reason for choosing this method is because the ligation methods are apt to be incomplete in assuring absolute removal of all ovarian tissue. When the broad blades of the angiotribe close upon the pedicle of the ovary, the ovary is crowded away from its seat and rises above the blades, thereby avoiding the possibility of leaving ovarian tissue behind. I have been particular in development of this point of technique in ovarian grafting, and in our experimental work with rabbits, my associate, Dr. L. A. di Zerega, has insisted that we must not allow even a particle of scraping from an excised ovary to fall back into the peritoneal cavity, because a single detached ovum might live in contact with the peritoneum long enough to invalidate the experiment. After removal of each ovary of Mrs. P., a slit was made through the peritoneum of the broad ligament on either side, parallel with the oviduct, and dorsad from the original seat of the ovary. Into each slit was placed a segment of ovary from Mrs. P., about one-half inch long, and one-fourth inch wide. A single catgut suture was placed in such a way as to hold each graft in place. Grafts are so placed that the cut surface of the ovary lies in contact with the cut surface of the broad ligament, in order that the nutrition of the graft may be maintained through the lymph circulation, pending the formation of new capillaries. Uncut surface of ovary is allowed to project into the free peritoneal cavity, to facilitate the free escape of ova. The uterus and oviducts of Mrs. W. were apparently normal. The ovaries removed from Mrs. W. were given to Dr. H. T. Brooks for microscopical examination, and he reported later that they were cirrhotic ovaries, distended with many small cysts, and containing no formed Graafian vesicles. Mrs. W. and Mrs. P. made customary recoveries from their operations.

Present at the operation upon Mrs. W. were Dr. H. J. Boldt of New York, and the hospital staff assistants, Dr. R. G. Treacy, now of New Haven, Conn.; Dr. W. H. Allee, now of Stamford, Conn.; and Dr. D. W. Cairns, now of New York.

On July 1, 1902, I received a letter from Dr. H. L. Barker, stating that for about a month after Mrs. W. returned from the hospital she felt well excepting for a continuance of the hot and cold flashes. On June 18, 1902, four months after the grafting, Dr. Barker wrote that Mrs. W. had at that date menstruated, menstruation lasting for five days. (Grafted patients have usually first menstruated at periods varying from two months to five months after the operation).

On March 28 of the following year (1903) Dr. Barker again wrote, stating that he had just seen Mrs. W. and obtained the following history: After the menstruation of June, 1902, she did not again menstruate until November 16 of the same year, when she flowed for one day. The next month, December 15, she flowed for four days, and menstruation after that became regular, with a flow for four or five days each period. The patient was "feeling perfectly well and happy."

Nothing more was heard from Mrs. W. until March 28 of the present year (1906) when I received a letter from Dr. Robert Lount of Hempstead, N. Y., stating that he had under his care a patient by the name of Mrs. H. W., who informed him that she was one of my ovarian grafting patients. He had on March 15, 1906, delivered her of a daughter weighing seven and one-half pounds, and mother and daughter were doing well, excepting for some manageable complications on the part of the mother. On April 4 I received a subsequent letter, stating that mother and daughter were doing well, and that the mother had a good flow of milk.

On April 14 I received a letter from Dr. H. L. Barker stating that he had examined Mrs. W. when she was about four months pregnant, but on account of her distance from his circuit of practice he had depended upon others for her care at parturition.

So far as I can judge we have in this case a well defined instance of heteroplastic ovarian grafting, followed by pregnancy, and the delivery of a normal child. If the pregnancy had appeared in one of my earlier grafting cases, when the ligature method for removal of ovaries was employed, I might have been a trifle skeptical about the nature of the case, but this pregnancy occurred after the adoption of a technique which was, to my mind, satisfactory in eliminating any weak points in our work.

Through all the varying ideas on the subject of ovarian grafting during the past few years I have felt that some one would eventually get a child out of some case of homoplastic grafting, as our experiments upon rabbits and other animals seemed to show that when the ovary of an animal was removed from the body, and then reintroduced in its original host, there was a tendency for the ovary to live, and to continue its function.

When, on the other hand, an ovary was grafted from one animal into another, the principle of intolerance of the tissues of one animal for the tissues of another animal came into play, and there was a tendency for the grafted ovary to degenerate or to become absorbed. It was noted, however, that there were various degrees of tolerance shown, and this seems to be an important point. One animal would absorb the ovaries of another animal very rapidly, while a third animal might carry the ovaries of a fourth for several months. In the case of Mrs. W. it is probable that we have an instance of tolerance for the tissues of Mrs. P. that may have amounted to complete tolerance. It is important for us to experiment along lines which will allow us to prepare animals for tolerating the tissues of each other, and I had planned to do this work, but in the midst of active practical professional work experiments go along slowly, and at the present moment I have material collected for an experiment in another field entirely, and which will demand every hour of spare time for a year at least.

At Cornell University Medical Laboratory I carried on one line of experiments, suggested by the known principles of hemolysis, with the assistance of Dr. James Ewing, Dr. George H. Ryder, and medical students, Messrs. E. D. Watkins, W. E. Drennan, and R. E. Pou. A series of rabbits were made immune to each other's serum, and their ovaries were then exchanged. These ovaries, as anticipated, were absorbed more rapidly than in animals not immune to each other's serum, but I wished simply to get the facts, in order to make use of the negative testimony.

In women I have, up to the present time, done homoplastic and heteroplastic ovarian grafting in fourteen cases, and in one case made a homoplastic graft of the fimbriated extremity of an oviduct in a case of fibronodular oviduct. The fimbriated end was fastened to the cornu of the uterus after excision of the nodular area, which included nearly all of the tube.

So far as I know there has been but one other case of conception among my grafted patients. This was in a case of homoplastic ovarian grafting, and the patient had a miscarriage at the third month. The case was not properly safeguarded for scientific report, and I have said little about it, and have not asked the profession to accept the testimony, but personally there is to my mind no reason for doubting the statements of the patient herself. I expect much of homoplastic grafting, but had about given up hope of obtaining pregnancy in heteroplastic grafting cases until we could prevent patients from absorbing the grafted ovary, and this case of the child of Mrs. W. came to me as a surprise. My efforts had turned toward perfecting methods for homoplastic grafting, for the purpose of avoiding the precipitate menopause, retaining the influence of the internal secretion of the ovary, and the normal sexual attitude of the patient, in selected cases in which for any reason both ovaries had to be removed. There seemed also to be a fair probability that pregnancy might sometimes occur.

The present case of heteroplastic grafting renews our hope that we may make fertile some women who have reached the menopause through disease, surgical operation, or possibly after normal menopause.

It will be interesting to note which parent the child of Mrs. W. most resembles. According to Haeckel, hereditary characteristics are due to cell memory, and the cell memory of an engrafted ovary may not be changed by the influence of its new host. It is fair to say, however, that the mother who bore the child is the real mother, as she furnished the nutrition for development of the child. It is somewhat important, aside from medical and from ordinary sociological interest, to place the status of this child, for in legal circles the word "descendants" will now come to have a larger meaning.

Concerning the matter of priority in this field of ovarian grafting, according to the *Index Medicus* the proposition of the idea, the description of the technique, and the report of cases were published in New York in the year 1895. The papers of Chrobak and Knauer followed in 1896.

Note.—A number of physicians with whom I have discussed the ovarian grafting case, state that they are sure that the ovaries were completely removed in cases in which menstruation continued. Questioning elicits the statement that such ovaries were removed by ligation methods, and that any portion of ovary remaining must have been "outside of the ligature, where it could receive no nutrition." This is the very point that is brought out in my experimental work in homoplastic grafting. A trifle of ovary outside of the ligature may receive enough lymph circulation by peritoneal contact to insure vitality, and it then becomes a homoplastic graft. A few cases of pregnancy have been reported in cases in which the ovaries were believed to have been removed. I think that these are, in fact, homoplastic graft pregnancies, and that they offer testimony in support of the theory that such pregnancies can occur readily enough if the oviducts are in condition to allow it. It is a question if menstruation can occur without influence upon the uterine ganglia of the internal secretion of that temporary organ, the corpus luteum. The employment of Tuffier's angiotribe or of methods of complete excision of the ovary, followed by suture instead of ligation, will insure complete removal of all ovarian tissue. I

do not remember to have had menstruation follow in any case in which either one of these methods was employed, unless grafts had been placed in the patient purposely.

616 Madison Avenue.

DISCUSSION.

Dr. C. G. Cumston of Boston referred to a recent article by Simon in which it was stated that microscopically two stages followed ovarian grafting. The ovary, during the first part of the time afer grafting the ovary, was only nourished by the interstitial fluids of the surrounding tissues. The cortex alone retained its vitality because the fluids could not reach the central portion of the gland. The medullary zone degenerated and disappeared while a cicatricial process became established. At the end of three or four months the ovary regained its normal circulation. The various ovarian elements showed modification in their histological structure, depending upon these different nutritive phases. The interstitial cells gave evidence of changes distinctly demonstrating the influence of the nutrition of the organ, and immediately after the graft they lost their power of differentiation, their boundaries disappeared, while their cell bodies considerably diminished in size. At the end of three months, when their nutrition was again normal, they again gave evidence of functional activity, and took on the appearance characteristic of their physiological state. In view of these facts a properly performed hetero-transplantation of an ovary might result in fecundation and pregnancy. The corpus luteum seemed to him to have considerable bearing on the question of future pregnancies after grafting had been done. Enough work had been done in the study of the relationship between the ovaries and metabolism generally to show that the ovary possessed an internal secretion, and that the influence of the ovary upon the genitalia and body as a whole was due to its action and not to the presence of sympathetic ganglia in the ilium of the gland. Fraenkel had concluded that the corpus luteum was a ductless gland and provided an internal secretion which presided over the nutrition of the uterus and the imbedding of the fertilized ovum, and in the absence of the fertilization of an ovum the internal lutein secretion excited the menstrual flow. He had used the corpus luteum of a cow as a therapeutic agent in cases of threatened abortion. The lutein substance was given to stimulate the growth of the trophoblast, upon which the process of successful imbedding depended. Fraenkel was satisfied with the results obtained in fifteen cases. So strongly had the view gained acceptance on the continent that the growing trophoblast was dependent upon the corpus luteum that clinicians were endeavoring to check conception by inhibiting the growth of the trophoblast by biochemical means, an antilutein extract being used for the purpose. The lutein tissue found around atresic follicles was held to be totally different in origin to that found within the theca of the true corpus luteum, some writers holding that the former was of connective tissue origin and the latter derived from epithelium. After extensive investigation Fraenkel decided that the *"glanda interstitiella"* was inconstant and very variable. It did not serve any important or general function in the human body. Subsequent to its rupture the corpus luteum was a hematoma lined by convolutions of lutein tissue. This tissue consisted of decidua-like cells held together by strands of connective tissue which carried vessels derived from a vascular tunic upon which the cells lay. This tunic was the theca interna; it surrounded the entire yellow body; it represented the old tunica fibrosa, but during the process of maturation of the Graafian follicle it had become highly vascularized and cellular. Internal to the lutein cells, and lying immediately upon them, was a membrane of variable thickness spoken of as the tunica propria by some and as the basal membrane by others. After discharge of the liquor folliculi and escape of the ovum the delicate new capillaries of the theca interna suffered disruption, blood escaping from them to fill the space previously occupied by the liquor folliculi. Thus a hematoma was formed, and it was in this stage that we generally met with a corpus luteum in the human ovary. He then described the process by which the corpus luteum disappeared. Islands of lutein tissue might escape destruction. In the study of lutein cysts a review of the degenerative stages of the ruptured corpus luteum had been almost barren of results. In studying the developmental processes which preceded rupture there were many difficulties. Of the sixty to eighty thousand primitive ova, only 150 to 200 were said to ripen, and it was a rare chance that provided one with a healthy human ovary. The ovum being a very delicate cell, it may suffer from preparation of the tissues for the microscope, and thus present appearances that were misleading. With regard to the origin of the lutein cell, it could be seen to arise from the connective tissue cells of the vascula theca interna outside the basal membrane of the stratum granulosum. He endeavored to show what was assumed to be a transition of the ordinary connective tissue cells into the large decidua-like cells which made up the large convolutions of the yellow body. He had worked on the assumption that the presence of lutein tissue in the theca interna of a small follicle was not of necessity a pathological phenomena, but that this tissue occurred during the healthy maturation of the follicle, and was in fact a progressive stage in its development. He was not prepared to say at what point in the development of the follicle the lutein cell was due to make its normal appearance, and in the same way at what time the granulosa normally broke up and no longer existed as a complete ring had not been demonstrated. He described lutein cysts and said that when a lutein cyst developed alongside of any other cystic space a complication cyst arose. In large islands of displaced lutein tissue lymphangiectases occurred, and by distention of these spaces nonepithelial lutein cysts arose. The possibility of transportation of ovaries was now demonstrated beyond a doubt. According to Schultz, both in young and in almost grown animals the ovaries became attached in their new position, whether this were the peritoneal cavity of the same female, of another female, or of a male, or of the same breed, or of a female of a different breed. On other females of the same breed transplanted ovaries of mammals could give off eggs and form corpora lutea; on males of the same breed such ovaries could develop ripe eggs up to forty-two days, and show such after one hundred and seventeen days; on females of other breeds such ovaries exhibited, in the first eight days, no difference from ovaries transplanted on females of the same breed; the mode of healing and development corresponded in the animals investigated to the phenomena described by Ribbert. and in ovaries transplanted to male eggs could be demonstrated in the root, and tube-formed processes crowding from the germinal epithelium downward into the tunica albuginea. At the same time changes took place that at least stimulated the embryonic condition. The transplanted ovarian tissue appeared not to be injuriously influenced either by the blood of the male or by any secretion or other influence from the testicles, at least in the short time covered by the experiments. Transplantation did not operate to produce any unusually rapid growth in ovarian tissue, as might possibly be expected. Dr. Cumston suggested that this subject be followed up by such experiments as implantation of ovaries in a female of another breed whose ovaries had been previously removed, and that breeding experiments be then carried on with the male of the same breed.

Dr. E. B. Cragin said that knowing Dr. Morris's care in experimental work and technique, he would be inclined to take his statements as facts simply because he said so. Still there were a number of tests to be applied to the case before accepting it, and he wished to speak on three things that Dr. Morris had laid particular stress upon, as follows: (1) The condition of the ovaries at the time of removal. It was reported that no Graafian follicles were found by the pathologist at the time of its removal, and, it seemed to him, more light should have been thrown upon this statement by the pathologist, especially regarding any developing Graafian follicles. (2) Symptoms presented at time of removal. It should be remembered that a woman might have absence of menstruation for a long period of time and yet not attain the menopause for a number of years later. There was no question but that the ovaries from a woman who was not menstruating, or who had not menstruated for seven years, might have at the time of removal a recent corpus luteum, showing that ovulation had taken place in spite of amenorrhea existing for seven years. Therefore more light upon this condition of the ovaries was needed in order to decide that the ovaries were unable or incapable of producing ova successfully. (3)

Method of removal of ovaries. He said that Dr. Morris laid stress upon the fact that the ovaries were removed by the angiotribe, but Dr. Cragin could not help feeling that possibly there was a little ovarian tissue in the stump. We did not know all that we should know regarding the anatomy of the ovarian tissues, but he believed that some of this ovarian tissue might have been left behind in spite of the use of the angiotribe. He said that a few years ago a woman came to him with an ovarian cyst on one side and a large cystic ovary on the other. Just before taking the anesthetic she said she would rather die than be made an "it." He removed both ovaries, and on examining the smaller one found that there were small portions which appeared healthy, and these he stitched in the broad ligament in the horns of the uterus. She was told that she was not an "it." She had menstruated irregularly ever since. Personally he believed that Dr. Morris's case would stand the test, and that he ought to be congratulated in contributing something to the cause of science.

Dr. Henry C. Coe expressed the highest admiration for the reader's successful work. The case was so carefully reported and the technique was so admirable that there seemed to be no question as to the scientific value of the communication. However, as criticism had been invited, the speaker wished to refer to certain lacunæ which should be noted. The report of the pathologist was not as thorough as could be wished in a case of such importance, as it was not made clear as to whether the ovaries were cirrhotic as the result of previous inflammation, or had simply undergone normal atrophy. The statement with regard to the condition of the Graafian follicles was vague and unconvincing. The age of the patient, and the fact that the uterus had not undergone the usual climateric atrophy, would seem to show that the case was one of suspension of the ovarian function rather than of premature menopause. Still the fact remained that the patient had not menstruated for a long period and that there was every reason to suppose that the flow would not have returned. Whether she had ovulated or not during this time was doubtful. The speaker called attention to the well-known type of amenorrhea in young women who became unusually obese, with or without the disturbance usually attending the normal climacteric. In such cases he had known menstruation to return and to recur regularly after it had long been suspended, as the result of reduction in weight, local stimulation with electricity, etc., though he could not recall an instance in which pregnancy had followed the renewal of the ovarian function. He had had no personal experience with ovarian grafting, but had frequently noted that in conservative operations upon the ovaries, where only a small portion of an ovary had been left, without disturbance of its vascular supply, that there was a decided tendency to atrophy or to cystic degeneration, and that menstruation was often irregular or ceased entirely. As the reporter had stated, the results after grafting had been so uncertain that there was so much the more reason to congratulate him on the brilliant result which had followed after so many previous discouragements. While the operation must have a very limited sphere, it seemed as if in this instance the highest judgment and skill had been exercised, both in the selection of a proper case and in the manner of handling it.

DR. HERMAN J. BOLDT said that while he was of the belief that it had not been established that the patient upon whom Dr. Morris operated had premature menopause, due to destruction of ovarian tissue—the pathologist's report being practically useless because no serial sections were made—the fact that there were no evidences of functionating ovarian tissue in the sections made by no means proved that there was no functionating tissue present; however that might be, the fact remained that, whether functionating or not, the ovaries were removed. Knowing of the case and seeing the operation performed, he did not think it likely that any ovarian tissue was left behind. It was most probable and most positive that the subsequent menstruation was established and caused by the ovarian tissue engrafted from one patient to the other. He said he was reminded of some cases he had had under observation, a mother and three sisters in one family, all four presenting from six months to two years between menstruations. Two of these sisters had families. This showed the possibility of not having menstruation and yet ovulation might occur. Personally he did not believe that one should sacrifice any organ apparently normal.

DR. ROBERT H. M. DAWBARN, referring to Dr. Morris's statement of future work he was to be engaged in, injecting the blood of one rabbit into the blood of another, with the idea of establishing tolerance, said that he had heard recently from Surgeon W. F. Arnold, U. S. N., who told him of a communication received from Metchnikoff's laboratory which had a direct bearing upon this work. It was found that a small amount of blood from the male animal injected beneath the abdomen of the female animal rendered her sterile, so far as that male animal was concerned. It was tried on dogs and other animals, and was found to be true in every instance. This, Dr. Dawbarn said, opened up a new field for both beneficial as well as dangerous work.

---

No surgical instrument is cheap unless it is made in the best way, and from the best possible material for the purpose. The way to buy instruments is to purchase them from the man who will charge least for something which he guarantees to be as good as the best in the market. There is nothing so expensive to the surgeon as knives and scissors that will not keep an edge, and locking instruments that will not stay locked after a brief period of usage.—*Medical Standard.*

